# Supplementary material for: The theory of planned behavior as a behavior change model for tobacco control strategies among adolescents in Botswana
Source: PLoS One. 2020 Jun 5;15(6):e0233462. doi: 10.1371/journal.pone.0233462 (PMC7274417; doi:10.1371/journal.pone.0233462)
Supplement: S1 Table — (DOCX) [file pone.0233462.s001.docx]

S1 Table. This is the S1 Table on Intension to smoke and having had a conversation with an adult about smoking, and they encouraged me to try smoking

S1 Table. Intension to smoke and having had a conversation with an adult about smoking, and they encouraged me to try smoking

| In the next 12 months, do you think you might smoke a cigarette? And In the past two months, I had a conversation with an adult about smoking, and they encouraged me to try smoking | | | | | |
| --- | --- | --- | --- | --- | --- |
|  | | | In the past two months, I had a conversation with an adult about smoking, and they encouraged me to try smoking | | |
|  |  |  | Yes | No | Total |
| In the next 12 months, do you think you might smoke a cigarette? | Yes | Count | 12 | 145 | 157 |
|  |  | % within In the next 12 months, do you think you might smoke a cigarette? | 8% | 92.% | 100.0% |
|  |  | % within In the past two months, I had a conversation with an adult about smoking, and they encouraged me to try smoking | 16% | 6 % | 6.2% |
|  | No | Count | 65 | 2112 | 2177 |
|  |  | % within In the next 12 months, do you think you might smoke a cigarette? | 3.0% | 97.% | 100.0% |
|  |  | % within In the past two months, I had a conversation with an adult about smoking, and they encouraged me to try smoking | 84% | 94 % | 85.8% |
| Total |  | Count | 77 | 2257 | 2334 |
|  |  | % within In the next 12 months, do you think you might smoke a cigarette? | 3.0% | 97% | 100.0% |
|  |  | % within In the past two months, I had a conversation with an adult about smoking, and they encouraged me to try smoking | 100.0% | 100.0% | 100.0% |
