# Supplementary material for: The theory of planned behavior as a behavior change model for tobacco control strategies among adolescents in Botswana
Source: PLoS One. 2020 Jun 5;15(6):e0233462. doi: 10.1371/journal.pone.0233462 (PMC7274417; doi:10.1371/journal.pone.0233462)
Supplement: Attachment — Submitted filename: Response to Reviewers.docx [file pone.0233462.s004.docx]

P.O. Box 82293

Gaborone

Botswana

Date: May 02, 2020

**To:**	Academic Editor

PLOS One

Attention Dr. Amir H. Pakpour

Dear Dr. Amir H. Pakpour

We are re-submitting our manuscript entitled “**The Theory of Planned Behavior as a behavior change model for tobacco control strategies among adolescents in Botswana**.” PONE-D-20-00392 for consideration by PLOS ONE after incorporating the comments we received. We appreciate this opportunity, as well as the thoughtful comments provided by the reviewers. Below are the comments we received and how we addressed them in our revision.

Reviewer #1: The revised manuscript is much improved, especially the English presentation. The authors also have answered all my prior comments in a satisfactory way. However, one minor issue needs to be fixed before acceptance. That is, the manuscript contains both British and American spelling (e.g., utilise; behavioral; behavioural). Please use Word function to check whether all the spellings are consistent and correct them in either British spelling or American spelling. Please do not use a mixture. Anyway, good work and I look forward to reviewing a new revision.

**We have edited the spellings to American English for example on page 4 we have changed the word behaviour to behavior and on page 5 we changed the word utilised to utilized. We have gone through the whole document using Word function to check for consistence in spellings.**

Thank you for your consideration. Please let us know if you have any questions or need further information.

Sincerely,

Mr Roy Tapera
